# Speech Perception in Bilateral Hearing Aid Users With Different Grades of Asymmetric Hearing Loss

**DOI:** 10.3389/fnins.2021.715660

**Published:** 2022-01-26

**Authors:** Ulrich Hoppe, Anne Hast, Thomas Hocke

**Affiliations:** ^1^Department of Audiology, ENT-Clinic, University of Erlangen-Nürnberg, Erlangen, Germany; ^2^Cochlear Deutschland GmbH & Co. KG, Hanover, Germany

**Keywords:** asymmetric hearing loss, efferent auditory system, afferent auditory system, auditory deprivation, hearing aids, speech recognition model

## Abstract

Hearing loss is associated with decreased speech perception as well as with changes in the auditory pathway. The effects of those changes on binaural speech perception with hearing aids are not yet fully understood. To provide further evidence on the functional changes of the auditory pathway, several speech perception tests (unilateral and bilateral, aided and unaided, in quiet, and in noise) were conducted in a population of 370 bilateral hearing aid users covering the entire range of the World Health Organization’s most recent classification of hearing loss. To characterize the effects of asymmetric hearing thresholds, a generalized linear model was used for regression analysis. The model revealed a detrimental effect of the poorer ears’ thresholds on both the unaided and the aided unilateral word recognition scores that were attained by the better ear. Moreover, aided binaural word recognition (in quiet and in noise) was affected to a degree that cannot be explained on the sole basis of bilateral summation. Thus, this study provides evidence that there is reorganization and altered functioning of the afferent and efferent auditory pathways due to asymmetric hearing loss. Consequently, more attention should be paid to provision with a hearing aid as early as possible, and separately for each ear.

## Introduction

Hearing loss is associated with a number of negative effects (Chia et al., 2007) and represents the fifth largest burden of disability (Vos et al., 2015). Additionally, according to the latest World Health Organization world report on hearing, hearing loss is the third largest cause of years with disability, and unaddressed hearing loss is estimated to impose a global cost of more than US $980 billion annually (WHO, 2021). For most people with chronic hearing loss, hearing aids (HAs) are the primary therapeutic option. HAs provide amplification and therefore better speech understanding in quiet and in noise. However, the degree of benefit varies substantially, and little is known about the actual causes of this variability. In consequence, the prevalence of HA use is rather low. The reported overall prevalence of HA use among adults varies between 9.7% (Sawyer et al., 2019) and 30% (Anovum, 2018; WHO, 2021). The actual use of HAs tends to increase with higher age, greater degree of hearing loss, the presence of comorbidities, and self-perceived limitations of hearing in everyday situations (Sawyer et al., 2019). Earlier classification of hearing loss by the WHO was based upon the pure-tone average (PTA) of the better-hearing ear throughout. Consequently, there was no recommendation for treatment of the worse ear. Recently, the WHO refined their classification and included an additional class for unilateral hearing loss. Additionally, the comment is made that “unilateral hearing loss can pose a significant challenge for an individual at any level of asymmetry. It therefore requires suitable attention and intervention based on the difficulty experienced by the person” (WHO, 2021). For several reasons (Lin and Reed, 2021), the most commonly used reference for hearing loss is the PTA. However, this measure certainly fails to reflect the full impact of hearing loss (e.g., Plomp, 1978). Therefore, standardized speech perception tests should complement pure-tone audiometry as an indispensable measure for individuals with hearing loss.

For many patients with hearing loss, its etiology is unknown. Though genetics play an important role, hearing decline generally starts in adult age and progresses over the years, either in steps or smoothly. Typically, hearing loss affects both ears in a similar way, and large asymmetries are rare. Asymmetric hearing loss has been estimated to affect 8.5–13.3% of the general population (Chia et al., 2007). The causes of asymmetric hearing loss are usually the same as for hearing loss in general; these include aging (age-related hearing loss), noise (noise-induced hearing loss), metabolic causes, genetic causes (genetic hearing loss), ototoxic drugs, viral infection, Ménière’s disease, and injuries to the head or the ear. However, some of these causes are more closely associated with symmetric hearing loss and others with asymmetric hearing loss.

In summary, for both symmetric and asymmetric hearing loss, causal therapies are often not available and bilateral HAs are recommended in order to obtain best hearing outcomes. Unfortunately, for hearing loss that progresses over the years, it is quite usual for the better ear to be provided with an HA later than the worse ear. This assumption is supported by monaural and binaural HA adoption rates in Germany of about 29 and 71%, respectively (Anovum, 2018). Hence, the better ear may remain understimulated, and this could lead to detrimental effects for hearing.

Recently, Kurioka et al. (2021) investigated speech perception in twenty-eight participants with asymmetric hearing loss. In particular, they measured word recognition scores at the highest just tolerable level (WRS_max_) for the participants’ ears separately. They found that the worse ears exhibited significantly reduced WRS_max_ when compared with ears of persons with symmetric hearing loss for given (equal) pure-tone hearing thresholds. They concluded that decreased auditory utilization of the worse-hearing ear may impair speech discrimination ability, and they identified a need for special rehabilitation. Their findings strengthen the deprivation hypothesis (Silman et al., 1984; Glick and Sharma, 2020). Silman et al. found poorer speech discrimination in the unfitted ear compared with the fitted ear. They postulated an auditory deprivation effect, indicating that reduced auditory input can induce adverse auditory plasticity through the central auditory pathway.

The aim of this study was to investigate speech-recognition scores with HAs at the conversation level, WRS_65_(HA), with reference to the most recent WHO classification. Another established reference measure for HAs and other technical interventions, WRS_max_ (Hoppe et al., 2014, 2016; Maier et al., 2018; McRackan et al., 2018; Franks and Jacob, 2019), was assessed. Furthermore, we measured the unaided speech perception threshold in quiet and word recognition scores in noise, and we also investigated the relationship between these routine clinical measures and the grade of asymmetry of hearing loss.

## Materials and Methods

### Ethics Approval Statement

This study was carried out in accordance with the Declaration of Helsinki. The protocol was approved by the Institutional Review Board at the University of Erlangen (No. 162_17Bc). All participants provided written informed consent before participation in the study.

### Patients

In this retrospective study, more than 2,000 HA examinations were screened; they were performed between August 2012 and September 2017 in the Erlangen ENT Clinic. Bilateral HA users with at least 3 months of HA experience, German as mother tongue, and a minimum age of eighteen were included. The exclusion criteria were a mean air–bone gap at 0.5, 1, 2, and 4 kHz of more than 5 dB and any technical defects of the patients’ HAs. Prior to measurements, otoscopy was performed and, if needed, cerumen was removed. The test results of 370 bilateral HA users (182 men, 188 women) aged 21–98 years (mean, 62.8 years; standard deviation, 16.2 years) were eligible for assessment.

### Measurements

#### Pure-Tone Air-Conduction

Thresholds were measured for frequencies between 0.125 and 8 kHz, and bone-conduction thresholds between 0.25 and 6 kHz, by using a standard clinical audiometer (AT900/AT1000 Auritec, Hamburg, Germany) with appropriate headphones (DT48, Beyerdynamic, Heilbronn, Germany). For each patient and ear, the pure-tone threshold was summarized by averaging the thresholds found at 0.5, 1, 2, and 4 kHz; these thresholds are referred to hereinafter as PTAs.

#### Speech Audiometry With Headphones

Speech recognition was assessed by using the Freiburg number test and the Freiburg word test (Hahlbrock, 1957). Both were conducted with monaural presentation using headphones. Multisyllabic (two-digit) numbers were used to measure the speech-recognition threshold (SRT) in quiet, i.e., the sound pressure level (SPL) that corresponds to a recognition score of 50%. Roughly, this level corresponds to the pure-tone loss at 500 Hz + 20 dB (Braun et al., 2012). For individuals with normal hearing, the SRT is at 18 dB SPL (Brinkmann, 1974).

This relationship is an established measure in German speech audiometry to check the consistency of audiometric findings. The Freiburg monosyllable test was used to measure speech-recognition scores at higher levels and in particular the maximum word recognition score (WRS_max_): Starting with 65 dB SPL, the presentation level was increased in increments of 5–15 dB until 100% speech intelligibility was attained, unless the sound level became intolerable for the user or the audiometer limit of 120 dB SPL was reached. The uncomfortable level corresponds to speech presentation at the lowest SPL that is no longer tolerated. For analysis, we used the variable “better ear WRS_max_,” defined as WRS_max_ for the ear with the better PTA.

#### Speech Audiometry in Free Sound Field

Additionally, the Freiburg monosyllable test was used to assess aided speech recognition in free sound field. Word recognition scores in quiet were determined with HA for the left and right ear separately, WRS_65_ (HA). For the monaural measurements, the contralateral side was adequately blocked with earplugs. Additionally, binaural measurements for WRS_65_ (HA) were performed in quiet and with masking noise at a signal-to-noise ratio of +5 dB.

Before performance of the speech perception measurements, HAs were checked by visual inspection and dynamic elicitation of acoustic feedback by shifting the earmolds, removing HA, and cupping the HA in hand. In addition, qualified personnel (HA acousticians) checked whether the type and model of the HA provided, and the amplification, were appropriate for the individual’s hearing loss. Amplification was checked by real-ear measurements (Aurical, Natus, Münster, Germany).

### Data Analysis

MATLAB software version R2019b (MathWorks, Natick MA, United States) was used for all calculations and figures. A generalized linear regression model (GLM) was applied to the data. For speech recognition scores, model data for sigmoid regression were calculated according to equation 1. For speech recognition threshold, a linear fit was derived by using equation 2.


(1) $$Score\left[\%\right]=\frac{100}{1+e^{-\left(\beta_{0}+\beta_{1}\cdot P\,T\,A+\beta_{2}\cdot A\,s\,y\,m\,m\,e\,t\,r\,y\right)}}$$

(2) $$S\,R\,T\,\left[d\,B\right]=\beta_{0}+\beta_{1}\cdot B\,E\,A+\beta_{2}\cdot A\,s\,y\,m\,m\,e\,t\,r\,y$$

BEA (better ear average) refers to the better-ear four-frequency average of the pure-tone thresholds. The asymmetry refers to the difference between BEA and the poorer-ear four-frequency average.

Any effects of hearing thresholds and asymmetric hearing loss on speech perception measures were considered significant if the *p*-value was below 0.05.

## Results

**FIGURE 1:**
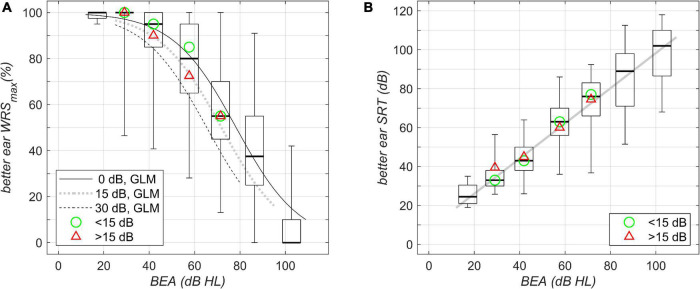
Unilateral unaided better-ear speech-recognition scores grouped by WHO classification of hearing loss (boxplots; left to right, Grades 0–6) and plotted against better-ear PTA. **(A)** Maximum word recognition score, WRS_max_, for the different WHO-grade groups as measured by the Freiburg monosyllable test. The boxplots summarize the WRS_max_ results; boxes show the medians and 1st and 3rd quartiles, while whiskers denote the 2.5 and 97.5 percentiles. The lines show the results of the fitted GLM: solid line, calculation performed for symmetric PTA (interaural difference 0 dB); dotted line, asymmetric PTA with interaural difference 15 dB; dashed line, asymmetric PTA with interaural difference 30 dB. **(B)** Speech-recognition score (SRT) in quiet as measured by the Freiburg multisyllable test. The solid line shows the result of linear regression; boxplots as in A summarize the SRT results. The green circle and the red triangle in **(A,B)** denote the median of the data with corresponding WHO grade for asymmetries below and above 15 dB, respectively.

**FIGURE 2:**
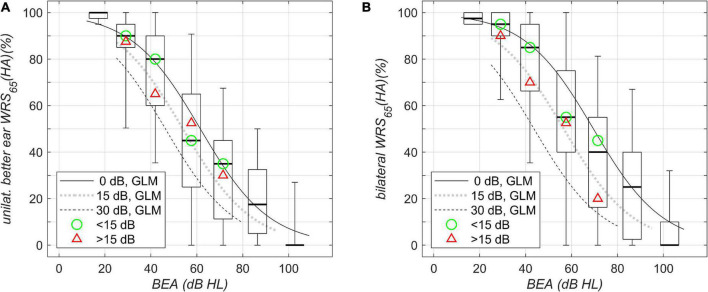
Aided speech-recognition scores in quiet grouped by WHO classification of hearing loss and plotted against better-ear PTA (BEA). **(A)** Unilateral WRS (HA)_65_. **(B)** Bilateral WRS (HA)_65_. Boxplots and solid/dotted/dashed lines as in Figure 1A. The green circle and the red triangle in **(A,B)** denote the median of the data with corresponding WHO grade for asymmetries below and above 15 dB, respectively.

**FIGURE 3:**
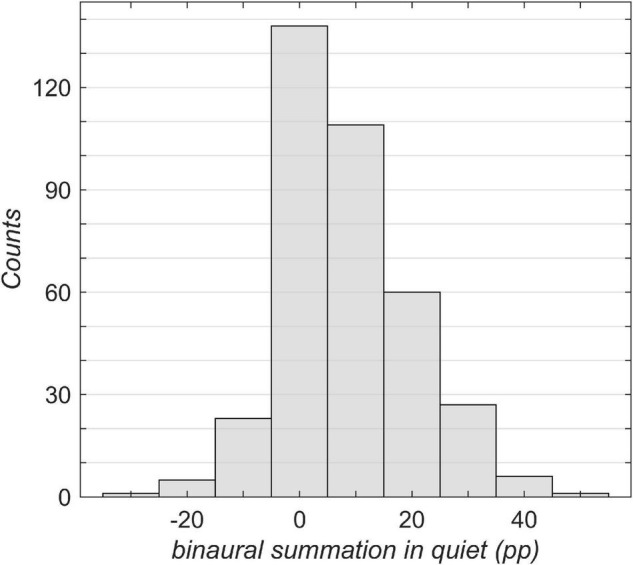
Overview of the study population broken down by binaural summation. The binaural summation was calculated as the difference in percentage points (pp) between the unilateral aided better-ear score (Figure 2A) and the bilateral aided score (Figure 2B). A negative value corresponds to binaural interference in which the bilateral score was poorer than the unilateral better-ear score.

**FIGURE 4:**
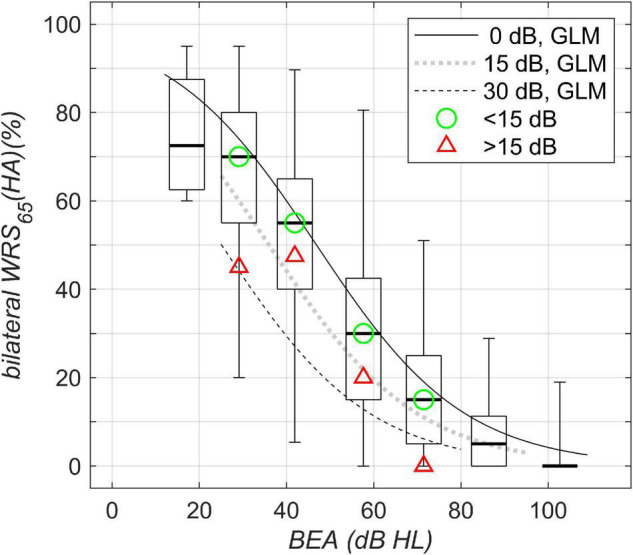
Bilateral aided speech-recognition scores in noise plotted against WHO classification of hearing loss. Boxplots and solid/dotted/dashed lines as in Figure 1A. The green circle and the red triangle denote the median of the data with corresponding WHO grade for asymmetries below and above 15 dB, respectively.

The results of the speech perception measurements are shown in Figures 1–4. Figure 1 shows the unaided speech perception scores in quiet, while Figures 2, 3 show the aided speech perception scores in quiet. Figure 4 refers to the aided speech perception in noise. The boxplots result from the grouping of hearing loss according to the WHO grade. In cases where there was a significant effect of asymmetric hearing loss on unilateral or bilateral scores, the results of the regression model are shown as examples for (i) symmetric hearing, (ii) for asymmetry of 15 dB, and (iii) for asymmetry of 30 dB. The characteristics of the study population are summarized in Table 1: age, WHO classification, and PTA asymmetry. Asymmetric hearing was similar across WHO grades up to Grade 6. Mean age was approximately the same in each WHO-grade group (Table 1), and no correlation was seen between age and the degree of hearing asymmetry (r_Spearman_ = 0.07, *p* = 0.18). For WHO Grade 6, the mean PTA difference was smaller because of audiometer limits; thresholds beyond the audiometer limits were set to the limit values.

**TABLE 1:** Patient characteristics.

WHO grade (PTA [dB])	Number of patients	Mean age [years]	PTA difference [dB]			
			Mean ± SD	No. of participants with PTA difference		
				0–10 dB	10–20 dB	> 20 dB
0 (< 20)	4 (1%)	63 ± 9	8 ± 5	2	2	0
1 (20–< 35)	51 (14%)	65 ± 10	8 ± 8	38	12	1
2 (35–< 50)	103 (28%)	65 ± 14	7 ± 6	68	31	4
3 (50–< 65)	97 (26%)	66 ± 16	7 ± 7	72	20	5
4 (65–< 80)	55 (15%)	61 ± 17	10 ± 8	35	15	5
5 (80–< 95)	32 (8.5%)	55 ± 17	8 ± 7	23	5	4
6 (≥ 95)	28 (7.5%)	49 ± 18	3 ± 3	25	3	0
All	370 (100%)	63 ± 16	8 ± 7	263 (71%)	88 (24%)	19 (5%)

### Unaided Speech Perception in Quiet

Figure 1A shows boxplots for WRS_max_ in dependence upon the BEA. BEAs were grouped according to WHO Grades 0–6. The scores show the largest variability of around 90 percentage points (pp) for WHO Grades 4 and 5. The model regression revealed a decrease in WRS_max_ of up to 20 pp; the greatest decrease was seen for an asymmetric hearing loss of 30-dB interaural PTA difference at a better-ear PTA of around 60-dB hearing loss (HL).

Figure 1B shows a corresponding analysis of SRT for multisyllables; here we found a large, continuously increasing variability with an increasing degree of hearing loss. The model regression did not reveal any significant effect of asymmetry on the better-ear SRT.

### Aided Speech Perception in Quiet

Figure 2A shows boxplots for aided word recognition score at a presentation level of 65 dB SPL, WRS_65_(HA), plotted against BEA. The scores show the largest variability, around 90 pp, for WHO Grade 3. For asymmetric hearing loss, with BEA around 60 dB HL and poorer-ear PTA around 90 dB HL, the model regression revealed a decrease of up to 24 pp in aided unilateral score. For the bilateral score (Figure 2B), the largest variability was again observed for WHO Grade 3. For symmetric hearing and PTAs of 60 dB HL, an improved score was found, compared with unilateral scores (53%), by about 12 pp and up to 65%. For asymmetric hearing loss with an interaural difference of 30 dB, there was already a decreased unilateral baseline performance (29%). Additionally, the model results not only revealed the absence of any binaural summation effect but also showed a slight binaural interference of 4 pp (25%). Therefore, the overall disadvantage for HA users with a better-ear PTA of 60 dB HL and an asymmetry of 30 dB adds up to 40 pp. With respect to asymmetry, the break-even for a binaural summation effect in quiet with BEA of 60 dB HL was found to be around 20 dB: for those cases, the two models yielded a unilateral score equal to the binaural score. Where the asymmetry was larger, binaural interference was dominant, while for binaural summation, a smaller asymmetry was found to be a precondition.

Figure 3 shows an overview of the binaural summation effect across all grades of hearing loss. A considerable part of the population (37%) has no significant binaural summation (i.e., below 5 pp). More than one-half (55%) of the population was assigned to positive categories, exhibiting a binaural summation effect. Less than one-tenth (8%) of the patients exhibited binaural interference of more than 5 pp.

### Aided Speech Perception in Noise

For bilateral speech perception in noise, Figure 4, the largest variability was found for WHO Grades 2 and 3. Owing to the test characteristic (with saturation and floor effects), for a BEA of 60 dB HL, the detrimental effect of asymmetric hearing was found to be up to 20 pp, while for a BEA of 40 dB HL, the detrimental effect of asymmetric hearing was found to be up to 31 pp. Both decrements are for asymmetric hearing of 30-dB side difference.

### Generalized Linear Regression Model

Table 2 summarizes the results for the GLM parameters. Parameters for sigmoid regression were calculated according to equation 1. For the linear fit according to equation 2, the GLM yielded a non-significant β_2_. Hence, we simplified equation 2 to equation 3:


(3) $$S\,R\,T\,\left[d\,B\right]=\beta_{0}+\beta_{1}\cdot P\,T\,A$$

**TABLE 2:** Parameters of the generalized linear regression models.

		Parameter	Estimate	Standard error	*t*-statistic	*p*	[β]	
Unaided scores in quiet	WRS_max_ (Figure 1A)	β_0_	5.60	0.13	43.4	<0.0001		
		β_1_	–0.0714	0.0018	–40.2	<0.0001	1/dB	PTA_better ear_
		β_2_	–0.0308	0.0044	–7.0	<0.0001	1/dB	Asymmetry
		7,400 observations, 7,397 error degrees of freedom, χ^2^-statistic vs. constant model: 2.5⋅10^3^, *p* < 0.0001						
	SRT (Figure 1B)	β_0_	7.97	1.803	4.4	<0.0001		
		β_1_	0.903	0.0307	29.4	<0.0001	1/dB	PTA_better ear_
		355 observations, 353 error degrees of freedom, F-statistic vs. constant model: 864, *p* < 0.0001						
Aided scores in quiet	Unilateral WRS_65_ (HA) (Figure 2A)	β_0_	4.09	0.10	39.5	<0.0001		
		β_1_	–0.0664	0.0017	–39.7	<0.0001	1/dB	PTA_better ear_
		β_2_	–0.0337	0.0040	–8.4	<0.0001	1/dB	Asymmetry
		7,400 observations, 7,397 error degrees of freedom, χ^2^-statistic vs. constant model: 2.5⋅10^3^, *p* < 0.0001						
	Bilateral WRS_65_ (HA) (Figure 2B)	β_0_	4.61	0.11	42.2	<0.0001		
		β_1_	–0.0663	0.0016	–40.2	<0.0001	1/dB	PTA_better ear_
		β_2_	–0.0572	0.0041	–14.0	<0.0001	1/dB	Asymmetry
		7,400 observations, 7,397 error degrees of freedom, χ^2^-statistic vs. constant model: 2.6⋅10^3^, *p* < 0.0001						
Aided scores in noise	Bilateral WRS_65_(HA) (Figure 3)	β_0_	2.76	0.10	28.7	<0.0001		
		β_1_	–0.0589	0.00172	–34.2	<0.0001	1/dB	PTA_better ear_
		β_2_	–0.0428	0.00427	–10.0	<0.0001	1/dB	Asymmetry
		7,400 observations, 7,397 error degrees of freedom, χ^2^-statistic vs. constant model: 1.8⋅10^3^, *p* < 0.0001						

In summary, the suprathreshold measures, unilateral WRS_max_, unilateral/bilateral WRS_65_ (HA) in quiet, and bilateral WRS_65_ (HA) in noise, depend on asymmetry. This was not found for the near threshold measure of the unilateral better-ear SRT.

## Discussion

Hearing outcomes for a large group of bilateral HA users were investigated within the context of routine clinical measurements. The population covered the degrees of hearing loss from WHO Grades 0–6. Outcome measures with and without HAs in quiet and in noise were found to depend significantly on the degree of asymmetry. For bilateral conditions, this is a well-known finding (Vannson et al., 2015; Jerger et al., 2017). However, the present study showed that even the unilateral scores in quiet on the better side are negatively affected by the degree of hearing loss on the contralateral (worse) side.

For the clinically relevant measures of the unilateral maximum word recognition score and the unilateral score with HAs, our results were similar to those of earlier studies (Hoppe et al., 2014). Unilaterally aided speech perception scores above 50% are typically found for hearing loss below 60 dB. All of the above studies referred to unilateral scores. Kronlachner et al. (2018) found a minimal effect of cognition on the success of HA provision. Additionally, in their population of 40 HA users, they found a deterioration of WRS_max_ with age. Müller et al. (2016) investigated age effects in elderly HA users and found effects for both measures [WRS_max_ and WRS_65_ (HA)] of the order of 10–20 pp. Regrettably, neither of these studies considered contralateral hearing or included binaural measurements.

### Effects of Asymmetric Hearing Thresholds on Better-Ear Speech Perception

For our study population, there was no correlation between age and degree of hearing asymmetry. Otherwise, the detrimental effect of age on speech perception would have been superimposed upon, or even have masked, the effect of asymmetric hearing. Most remarkably, even for unilateral WRS_max_, there were effects of the order of 20 pp. The PTA range in which this effect was the greatest is obviously determined by the ceiling effects of the speech material used and the presentation levels applied. WRS_max_ is typically measured near the discomfort level, while SRT is measured at a low level; the different presentation levels are probably the root cause of the different findings; as for SRTs in quiet, the asymmetry did not show significant effects in our study population.

For the purpose of simplification, the impact of hearing loss can be attributed in terms of functionality to two different components (Plomp, 1978; Plomp and Mimpen, 1979): (i) The attenuation component simply describes the effect of weakened sound perception due to the sensorineural component of hearing loss. This component should be easily compensated for by acoustic amplification. (ii) The distortion component refers to the loss of dynamic, frequency dependence of hearing loss, and the loss of temporal processing.

Complementary to this functional description of the impact of hearing loss, there is pathophysiological classification for hearing loss, which was originally applied to different types of presbyacusis. Schuknecht (1964) and Johnsson and Hawkins (1972) proposed for presbyacusis the terms sensory, metabolic, mechanical, vascular, and neural; for the latter, we prefer the term “central,” reserving “neural presbyacusis” for hearing loss due to the degeneration of the cochlear nerve. Within this classification, sensory presbyacusis is equivalent to the attenuation component. All the other types are summarized by the distortion component. Commonly, age-related hearing loss (presbyacusis) means hearing loss in the elderly. However, the term does not refer exclusively to aging of the auditory pathway; it can be interpreted as having a much broader meaning. Consequently, it includes the cumulative, genetically determined effects of aging, and this may also include possible damage to the auditory system caused by environmental noise (Johnsson and Hawkins, 1972; Schacht and Hawkins, 2005). One may therefore apply the above classification to the findings in our population, i.e., HA users with sensorineural hearing loss.

One possible cause for the observed detrimental effect of asymmetric PTA on unilateral better-ear scores could be the deprivation of the contralateral ear (Kurioka et al., 2021). Our data suggest that this deprivation may have an effect not only on the afferent auditory pathway but also on the efferent system. For the WRS_max_ at the near-uncomfortable presentation level, we found an effect, but not for the SRT, which is measured at near-threshold levels. The effects of the efferent system are believed to begin significantly above the hearing threshold if they are to be measurable. Hence, it is reasonable to consider that WRS_max_ is influenced by asymmetry while SRT is not. Therefore, one may at least partially assign the lower scores for the asymmetric PTA cases to a compromised efferent system in those patients. Unfortunately, the objective assessment of such effects by otoacoustic emission in individuals with hearing impairment presents a Gordian knot. Following the discovery of otoacoustic emissions (Gold, 1948; Kemp, 1978), their measurement was soon found useful for the objective assessment of effects that can be assigned to the efferent auditory pathway (Guinan, 2018; Lopez-Poveda, 2018; Fuchs and Lauer, 2019; Lauer et al., 2021). In patients with significant hearing loss, this approach is not possible owing to the lack of measurable otoacoustic emissions. Even though ultimate evidence is still lacking, we hypothesize that the missing efferent mechanisms result in deteriorated speech recognition. Following the functional description by Plomp (1978), these effects of impairment can be attributed to an increased distortion component of hearing loss.

The GLM revealed detrimental effects of asymmetric hearing, namely, the effect of the poorer-ear PTA on speech perception by the better ear. However, such a model does not permit a more detailed analysis. It remains unclear whether those effects can be attributed exclusively to interaural asymmetry. For higher degrees of hearing loss in the better ear, the poorer-ear PTA is subject to “numerical” saturation effects due to the limits of the audiometer. It is reasonable to assume that higher degrees of hearing loss have a detrimental effect on the efferent system as well.

### Bilateral Speech Perception

The binaural summation effect might be regarded as a result of the loudness increase from one to two ears for unimodal bilateral and symmetric listening (Christen, 1980; Rawool and Parrill, 2018). It is well known that binaural summation does not occur in all HA users (Arkebauer et al., 1971; Allen et al., 2000). Arkebauer et al. (1971) found a detrimental interaction between ears exhibiting bilateral asymmetric hearing loss, later referred to a binaural interference. They already highlighted the observation that in “cases with bilateral hearing loss, candidacy for binaural amplification should be determined from each ear independently, and the combined effect of both aids.” Our study design did not allow for determination of an effect of age on binaural interference. A corresponding matching of HA users was not possible. The model revealed a detrimental effect of asymmetry on binaural summation in quiet and noise. Trivially, asymmetry is equivalent to poorer PTA on the poorer ear and is therefore less surprising than a detrimental effect of asymmetry on unilateral better-ear scores. However, as the example of HA users with better-ear PTA around 60 dB impressively illustrates (Figure 2), the two disadvantages add up. For asymmetric hearing loss, both symptoms (the already decreased perception on the better ear and the missing binaural summation, or even binaural interference) might be caused by afferent and efferent deprivation.

### Limitations of the Study

The retrospective approach of this study certainly needs confirmation from prospective studies. Even though the number of HA users included was relatively high, the large variability in age, in hearing loss, and in experience with HAs prevents a more sophisticated evaluation. The GLM is *per se* an average-based model. The average model output was based on many different patients with highly variable progress of hearing loss, in some cases differing very strongly between the ears of the same study participant. Attempts at in-depth analysis, especially if retrospective, can easily result in an overfit of a model if it includes too many parameters. Even though in our study the GLM yielded significant effects of asymmetric PTA, it has to be stressed that these findings are preliminary owing to the retrospective study design.

According to clinical routine at our institution, HA users undergo unilateral assessment of speech perception on each side in quiet. Speech in noise is assessed bilaterally only. Altogether, each HA user routinely undergoes six different speech tests. Consequently, one cannot exclude the possibility that in some patients fatigue effects may have played a role, and thus increased the variability in the data. Probably the most important shortcoming of this study with respect to the hypothesized root causes of the effect of asymmetry, namely deprivation of afferent and efferent pathways, is the lack of detailed knowledge about the progress of individuals’ hearing loss. In a first attempt, it seems to be obvious to assign larger detrimental effects of asymmetry to a longer period of asymmetry. However, although this may fit in better with what we know so far, one cannot exclude the possibility that, after asymmetry has set in, reorganization of the auditory pathway may help in overcoming such detrimental effects. Recent findings in unilaterally deaf patients indicate such reorganization within as little as 1 year (Müller et al., 2017). The present study is a snapshot of a typical clinical population of HA users, and as such, it does not reveal deeper insights into the time course and the direction of detrimental effects in patients with and without asymmetric hearing loss. However, in view of the large effects of asymmetric PTA on speech perception that we have observed, further and deeper investigations of auditory deprivation effects are needed, particularly with reference to the efferent innervation of the better ear.

### Clinical Consequences for the Treatment of Asymmetric Hearing Loss

The results of this study suggest strongly that early treatment of hearing loss may be beneficial, even if the hearing loss is asymmetric and the prescription of an HA for both ears may not be considered urgently needed by the patient. Reimbursement criteria should reflect the detrimental effects of asymmetric hearing loss such that “in cases with bilateral hearing loss, candidacy for binaural amplification should be determined from each ear independently, and the combined effect of both aids” (Arkebauer et al., 1971).

## Conclusion

In a population of hearing-aid users, including symmetric and asymmetric PTA, the asymmetry exerts a detrimental effect on both unaided and aided word recognition by the better ear. Also, the binaural speech perception with HAs worsens with increasing asymmetry. This decrease exceeds the limits of a missing binaural summation by far. More attention has to be paid to provision of an HA as early as possible. There is an evident need for more research on the short- and long-term effects of asymmetric hearing on the afferent and efferent auditory pathways in individuals with hearing impairment.

## Data Availability Statement

The raw data supporting the conclusions of this article will be made available by the authors, without undue reservation.

## Ethics Statement

The studies involving human participants were reviewed and approved by Ethik-Kommission der Friedrich-Alexander-Universität Erlangen-Nürnberg. The patients/participants provided their written informed consent to participate in this study.

## Author Contributions

UH: conception and design, acquisition and interpretation of data, and final approval. AH: revision and final approval. TH: drafting and design, figure preparation, and final approval. All authors contributed to the article and approved the submitted version.

## Conflict of Interest

TH was employed by Cochlear GmbH & Co. KG, Germany. The remaining authors declare that the research was conducted in the absence of any commercial or financial relationships that could be construed as a potential conflict of interest.

## Publisher’s Note

All claims expressed in this article are solely those of the authors and do not necessarily represent those of their affiliated organizations, or those of the publisher, the editors and the reviewers. Any product that may be evaluated in this article, or claim that may be made by its manufacturer, is not guaranteed or endorsed by the publisher.
